# The Effect of Therapeutic Blockades of Dust Particles-Induced Ca^2+^ Signaling and Proinflammatory Cytokine IL-8 in Human Bronchial Epithelial Cells

**DOI:** 10.1155/2015/843024

**Published:** 2015-11-10

**Authors:** Ju Hee Yoon, Sung Hwan Jeong, Jeong Hee Hong

**Affiliations:** ^1^Department of Physiology, Graduate School of Medicine, Gachon University, 191 Hambakmeoro, Yeonsu-gu, Incheon 406-799, Republic of Korea; ^2^Division of Pulmonary, Allergy and Critical Care Medicine, Gachon University, Gil Medical Center, Incheon 405-706, Republic of Korea

## Abstract

Bronchial epithelial cells are the first barrier of defense against respiratory pathogens. Dust particles as extracellular stimuli are associated with inflammatory reactions after inhalation. It has been reported that dust particles induce intracellular Ca^2+^ signal, which subsequently increases cytokines production such as interleukin- (IL-) 8. However, the study of therapeutic blockades of Ca^2+^ signaling induced by dust particles in human bronchial epithelial cells is poorly understood. We investigated how to modulate dust particles-induced Ca^2+^ signaling and proinflammatory cytokine IL-8 expression. Bronchial epithelial BEAS-2B cells were exposed to PM_10_ dust particles and subsequent mediated intracellular Ca^2+^ signaling and reactive oxygen species signal. Our results show that exposure to several inhibitors of Ca^2+^ pathway attenuated the PM_10_-induced Ca^2+^ response and subsequent IL-8 mRNA expression. PM_10_-mediated Ca^2+^ signal and IL-8 expression were attenuated by several pharmacological blockades such as antioxidants, IP_3_-PLC blockers, and TRPM2 inhibitors. Our results show that blockades of PLC or TRPM2 reduced both of PM_10_-mediated Ca^2+^ signal and IL-8 expression, suggesting that treatment with these blockades should be considered for potential therapeutic trials in pulmonary epithelium for inflammation caused by environmental events such as seasonal dust storm.

## 1. Introduction

Meteorological and seasonal dust events in eastern Asia negatively impact humans and the ecosystem [1, 2]. These dust storms travel long distances, which increase the likelihood that they contain airborne particles, chemical components, and/or bacterial and fungal mediators, all of which can reach distant communities through dry deposition [3–5]. Because dust events create an atmospheric bridge over continents and oceans, numerous studies investigating the respiratory effects of dust particles have been conducted in the past few decades [6–9].

Bronchial epithelial cells are the first physical barrier of defense against exogenous stimuli, such as dust, allergens, pollen, and osmotic molecules. Thus, they are an important defense protecting the airway from respiratory pathogens. There are several evidences to address the airway pathology that repetitive exposure of mice to airborne dust particles induces lung inflammation [10, 11]. Furthermore, mineral-dust particle exposure was significantly associated with exacerbation of asthma in children [9]. Ambient particulate matter (PM) induces cytokine expression, including interleukins (ILs), leukemia inhibitory factor (LIF), and granulocyte macrophage colony-stimulating factor (GM-CSF) in human bronchial epithelial cells [12].

PM_10_, particulate matter with a diameter of less than 10 *μ*m, promotes fibrosis and intracellular reactive oxygen species (ROS) [2]. ROS causes oxidative damage to cellular components. Dust particles also induce transforming growth factor-*β*1 (TGF-*β*1) via ROS in bronchial epithelial cells. Recently, the effects of dust particle-induced oxidative stress were associated with immune function in alveolar macrophages and lung tissue [13, 14]. Ultimately, these oxidative stresses destroy the tight junctions of airway epithelial cells through activation of the transient receptor potential melastatin 2 (TRPM2) and cause transcription of major inflammatory genes [15]. The nonselective cationic channel protein TRPM2 is a pivotal regulator of Ca^2+^ signaling, influencing cell function and survival. The enzymatic domain of TRPM2 binds NAD^+^-metabolite ADP-ribose (ADPR), a process induced by poly(ADP-ribose) polymerase 1 (PARP-1); this binding results in channel activation, which facilitates Ca^2+^ movement into the cell and affects membrane potential [16]. Intracellular Ca^2+^ plays a pivotal role as a second messenger in the regulation of a diverse range of cellular functions such as muscle contraction, secretion, synaptic plasticity, cell proliferation, and apoptosis. Excess cytosolic Ca^2+^ due to the activation of TRPM2 leads to physiological and pathological responses including chemokine production, neutrophil migration [17], and neurovascular dysfunction [18].

Interleukin-8 (IL-8), a neutrophil-attracting cytokine and activating peptide, is well known to be expressed in bronchial epithelial cells. Furthermore, some have suggested that IL-8 migrates toward an injured site or it is produced more at sites of injury [19] and that it plays a role in the pathogenesis of allergic inflammation of bronchial asthma [20]. Lipopolysaccharide- (LPS-) induced cytokine production in human monocytes, such as IL-8 production, is principally mediated by excessive Ca^2+^ entry via TRPM2, and TRPM2 also promotes inflammatory neutrophil infiltration [17, 21].

It has been reported that dust particles induced Ca^2+^ signals [22, 23] and increased ROS levels induced by dust particles enhanced fibrogenic and inflammatory mediators [2, 12]. However, the identities of the Ca^2+^ signaling pathway activated by dust particles remain unclear in human airway epithelial cells. In the present study, we have investigated the direct effects of dust particles PM_10_ on Ca^2+^ signaling and responses to various treatments in Ca^2+^ signaling and proinflammatory cytokine IL-8 mRNA expression in human bronchial epithelial cells.

## 2. Materials and Methods

### 2.1. Reagents

BEAS-2B cells, derived from human bronchial epithelial cells, were purchased from American Type Culture Collection (Rockville, MD). Fura-2/AM was purchased from Teflabs (Austin, TX). Pluronic F-127 (20% in dimethyl sulfoxide) and 1,2-bis(2-aminophenoxy)ethane-N,N,N′,N′-tetraacetic acid tetrakis, acetoxymethyl ester (BAPTA, AM) were from Invitrogen (Carlsbad, CA). U73122 and its analog U73343 were from Tocris (Minneapolis, MN). Dulbecco's Modified Eagle's Medium (DMEM), penicillin-streptomycin, trypsin-ethylenediaminetetraacetic acid (EDTA), phosphate-buffered saline (PBS), and fetal bovine serum (FBS) were from Invitrogen (Carlsbad, CA). TRPM2 rabbit polyclonal antibody was from Abcam (San Francisco, CA). Monoclonal *β*-actin antibody, cyclopiazonic acid (CPA), nifedipine, lanthanum chloride (LaCl_3_), clotrimazole (CLZ), 3-aminobenzamide (3-AB), N-(p-amylcinnamoyl)anthranilic acid (ACA), 2-aminoethoxydiphenyl borate (2-APB), chlorpromazine (CLP), caffeine, dithiothreitol (DTT), N-acetylcysteine (NAC), diphenyleneiodonium (DPI), and all other chemicals not mentioned here were from Sigma-Aldrich (St. Louis, MO).

### 2.2. Asian Dust Particles (PM_10_) Sampling, Analysis, and Measurement of Particles Size

Air samples were collected in Incheon City, South Korea, and analyzed as described previously [2]. PM_10_ suspensions consisted of (%) 48 SiO_2_, 12 AL_2_O_3_, 5 Fe_2_O_3_, 5 CaO, 4 K_2_O, 2.37 MgO, 2 Na_2_O, and 1 TiO_2_. PM_10_ suspensions were sterilized at 360°C for 30 min to remove adhered microorganisms or other organic materials and stored at −20°C until use. The level of LPS in heated PM_10_ was below the detection limit (0.005 EU/mL) by a Limulus Amebocyte Lysate Assay Kit (BioWhittaker, MD). Thus, the effect of LPS of heated PM_10_ was not considered in this study. Particle size was measured in a Zeta potential and particle size analyzer (ELSZ-1000, Otsuka Electronics, Japan).

### 2.3. Cell Culture

The BEAS-2B cells were incubated at 37°C in a humidified cell culture incubator composed of 95% air and 5% CO_2_ in DMEM containing 10% FBS with antibiotics (100 U/mL penicillin and 100 *μ*g/mL streptomycin). When the cell culture reached 80% confluence, the cells were treated with trypsin/EDTA for 2 min to disperse and then transferred to new culture dishes or to glass coverslip-covered dishes for Ca^2+^ measurement.

### 2.4. Measurement of Intracellular Ca^2+^

BEAS-2B cells were cultured on cover glass, incubated with 4 *μ*M of Fura-2, AM in the presence of 0.05% Pluronic F-127 for 15 min in physiological salt solution (PSS) at room temperature in the dark, and washed with PSS for 10 min. The PSS contained the following [in mM]: 140 NaCl, 5 KCl, 1 MgCl_2_, 1 CaCl_2_, 10 HEPES, and 10 *D*-glucose, titrated to pH 7.4. For the 0 Ca^2+^ extracellular solution, CaCl_2_ was replaced with 10 mM ethylene glycol-bis(2-aminoethylether)-N,N,N′,N′-tetraacetic acid (EGTA). Change in intracellular Ca^2+^ was measured by the intensity of fluorescence with excitation wavelengths of 340 and 380 nm, respectively, and an emission wavelength of 510 nm. All results are reported as the fluorescence ratio, calculated as the ratio of *F*_340_/*F*_380_. Fluorescence was monitored with a charge-coupled device camera (Photometrics, Tucson, AZ) attached to an inverted microscope (Olympus, Japan) and analyzed with a MetaFluor system (Molecular Devices, PA). Fluorescence images were obtained at 1-second intervals. Background fluorescence was subtracted from the raw background signals at each excitation wavelength. The number of cells used in ΔCa^2+^ analysis was counted by Integrated Morphometry Analysis of MetaMorph software (Molecular Devices).

### 2.5. Imaging of ROS Signal

BEAS-2B cells were incubated on a cover glass in the presence or absence of 50 *μ*g/mL PM_10_ in PSS containing 10 *μ*g/mL ROS fluorescence probe 5-(and-6)-choloromethyl-2′,7′-dicholorodihydrofluorescin diacetate (CM-H2DCFDA, Invitrogen) for 5 min and washed for 5 min in PBS. H2DCFDA fluorescence was measured using a confocal laser-scanning microscope (Leica, Buffalo, NY) by excitation at 488 nm and measuring the emitted light at 525 nm. H2DCFDA fluorescence was collected for six different regions in each image, and the signal was normalized to that at the beginning of the experiment.

### 2.6. Reverse Transcription-Polymerase Chain Reaction (RT-PCR)

Total RNA was extracted from BEAS-2B cells using the TRIzol Purification System (Invitrogen) according to the manufacturer's instructions. Total RNA was amplified according to the manufacturer's protocol using AccuPower RT PreMix (Bioneer, South Korea). cDNA was amplified by PCR with HiPi Thermostable DNA polymerase (Elpis, South Korea) and nested primers. The forward and reverse GAPDH primers were GTCGGAGTCAACGGATT and GCCATGGGTGGAATCATA, respectively. The forward and reverse IL-8 primers were ATGACTTCCAAGCTGGCCGTGGCT and TCTCAGCCCTCTTCAAAAACTTCT, respectively. cDNA PCR began with a denaturation step at 95°C for 5 min, followed by 35 cycles of 1 min at 95°C, 1 min at 56°C (GAPDH) and 58°C (IL-8), 1 min at 72°C, and a final step at 72°C for 10 min. The PCR products were electrophoresed on 1% agarose gels. Expression levels of all PCR products were subtracted from those of a GAPDH loading control by measuring the intensity of PCR products with MetaMorph software (Molecular Devices).

### 2.7. Western Blotting

Cells were cultured and cell lysates were prepared in lysis buffer (contained 20 mM Tris, 150 mM NaCl, 2 mM EDTA, 1% Triton X-100, and a protease inhibitor mixture) by passing 10–12 times through a 27-gauge needle after sonication. The lysates were centrifuged at 11,000 ×g for 20 min at 4°C, and protein concentration in the supernatants was determined. Proteins were denatured by heating in SDS sample buffer at 37°C for 30 min. The 30 *μ*g heated protein samples were subjected to SDS/PAGE and subsequently transferred to methanol-soaked polyvinylidene difluoride (PVDF) membranes. Transferred proteins on PVDF membranes were visualized with TRPM2 and *β*-actin antibodies by enhanced luminescent solution (Thermo scientific).

### 2.8. Statistical Analysis

Data are reported as mean ± SE. Statistical significance was determined by analysis of variance in each experiment using the paired Student's *t*-test.

## 3. Results

### 3.1. Size Distribution of PM_10_ Dust Particles and PM_10_-Induced Ca^2+^ Signal by Extracellular Ca^2+^ and ROS Signal in Human Bronchial Epithelial BEAS-2B Cells

**Figure 1:**
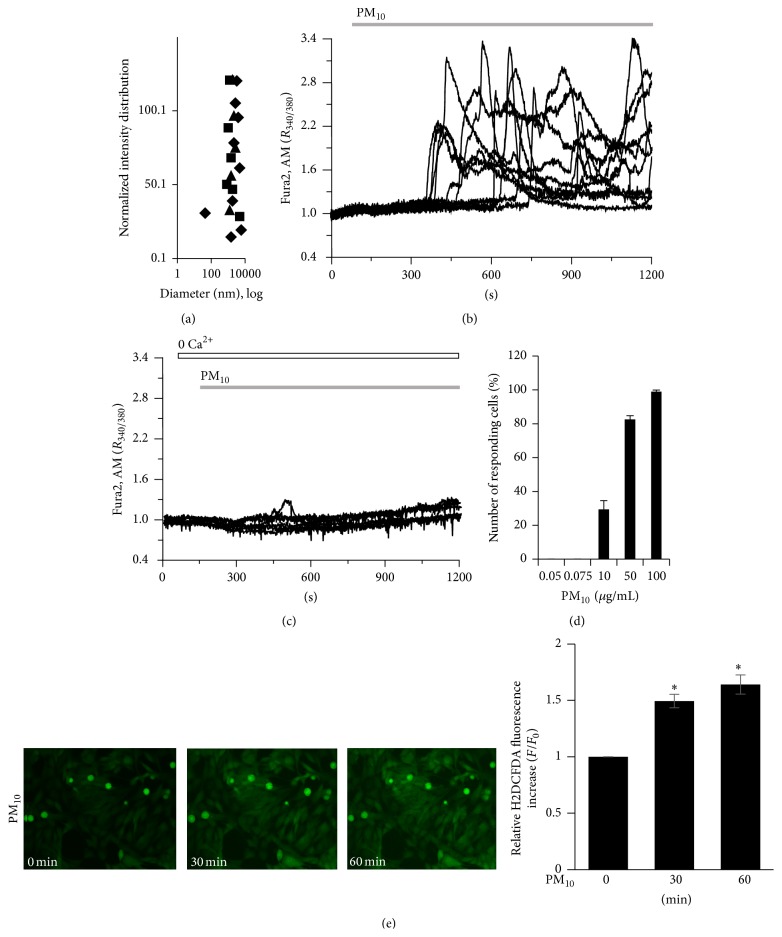
Size distribution of PM_10_ dust particles and PM_10_-induced Ca^2+^ signaling and ROS signal in human bronchial epithelial BEAS-2B cells. (a) Diameter of PM_10_ dust particles. Three kinds of shape represent independent applications to the particle analyzer. (b) Changes in [Ca^2+^]_i_ induced by 50 *μ*g/mL PM_10_ in 1 mM Ca^2+^medium and (c) in Ca^2+^-free medium. Top bars on trace panels indicate the extracellular solutions with which the cells were treated. (d) Number of responding cells in different dose of PM_10_ at 0.05, 0.075, 10, 50, and 100 *μ*g/mL PM_10_. (e) Time-dependent ROS signal after exposure to 50 *μ*g/mL PM_10_. The fluorescence of H2DCFDA was subsequently visualized by confocal laser scanning microscopy and quantified, and the relative intensities were calculated by setting the fluorescence intensity of control cells at 0 min to a value of 1. Data are represented as mean ± SE. ^*∗*^ *P* values <0.01 were considered significant.

Particles were filtered to 10 *μ*m and characterized as PM_10_, which included submicroparticles less than 900 nm in size as well as fine nanoparticles less than 100 nm in size (Figure 1(a)). For the PM_10_-induced Ca^2+^ signal, intracellular Ca^2+^ showed an oscillatory pattern in cells exposed to PM_10_. Ca^2+^ response showed 2~3 min latency since PM_10_ was applied, suggesting that PM_10_ signaling events occur prior to Ca^2+^ influx (Figure 1(b), *n* = 111 cells). To characterize the source of Ca^2+^, cells were stimulated by PM_10_ in the presence or absence of extracellular Ca^2+^. The PM_10_-induced Ca^2+^ signal was dramatically reduced in the absence of extracellular Ca^2+^ (Figure 1(c), *n* = 140 cells). Number of the responding cells increased PM_10_ concentration-dependently (Figure 1(d), 0% for 0.05 and 0.075 *μ*g/mL, 29.4 ± 5.21% for 10 *μ*g/mL, 82.6 ± 2.11% for 50 *μ*g/mL, and 99 ± 0.91% for 100 *μ*g/mL; *n* = 41,35,34,46, and 62 cells, resp.) even though this oscillation did not show periodic pattern. Because 50 *μ*g/mL PM_10_ did not induce cell death (data not shown) and generally produced reliable Ca^2+^ oscillations, we selected this concentration to analyze the mechanism by which PM_10_-stimulated Ca^2+^ signaling occurred in subsequent experiments. Cells were loaded with H2DCFDA to determine the extent of dust particles-induced ROS signal. Fluorescence was increased in a time-dependent manner after the application of dust particles (Figure 1(e), *n* = 6 regions of interest, four independent experiments). These observations indicated that PM_10_ induced an intracellular Ca^2+^ signal which was mediated by the availability of Ca^2+^ influx and ROS signal increase in bronchial epithelial cells.

### 3.2. The Additive Effect of PM_10_-Induced Ca^2+^ Signal in Store-Operated Ca^2+^ Influx Machinery

**Figure 2:**
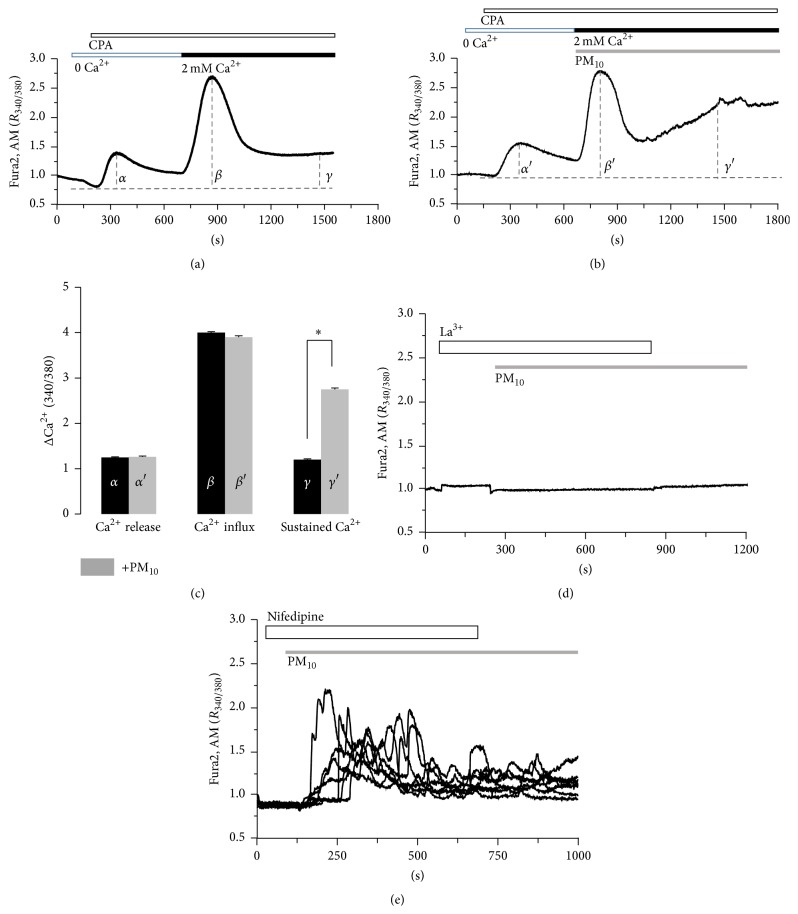
The additive effect of PM_10_-induced Ca^2+^ signal in store-operated Ca^2+^ influx machinery. The 50 *μ*M CPA-induced Ca^2+^ increase in the presence of 2 mM extracellular Ca^2+^ in the (a) absence or (b) presence of 50 *μ*g/mL PM_10_. The top bars on trace panels indicate the type of extracellular solutions applied to the cells and traces are the reading from an experimental average. (c) Analysis of CPA-induced Ca^2+^ release (*α*), influx (*β*), and sustained Ca^2+^ (*γ*) was determined using *F*_340/380_ fluorescence ratios from baseline (dotted). Results are means ± SE from three independent experiments. ^*∗*^ *P* values <0.01 were considered significant. (d) 100 *μ*M Lanthanum chloride (La^3+^), a nonselective cation inhibitor. Trace is the reading from an experimental average. (e) PM_10_-induced Ca^2+^ increase in the presence of 10 *μ*M nifedipine, an inhibitor of voltage dependent L-type Ca^2+^ channels. Each trace is the reading from a single cell.

To determine whether the PM_10_-induced Ca^2+^ signal was modulated by store-operated Ca^2+^ influx machinery, cells were treated with cyclopiazonic acid (CPA) in the absence of extracellular Ca^2+^ to deplete Ca^2+^ stores in the endoplasmic reticulum, and 2 mM extracellular Ca^2+^ was then applied. PM_10_-induced Ca^2+^ was additive effect on the store-depleted Ca^2+^ influx signal (Figures 2(a) and 2(b), *n* = 130 and 144 cells, resp.) with a sustained Ca^2+^ level (Figure 2(c)). To verify the PM_10_-induced Ca^2+^ increase due to the influx of extracellular Ca^2+^, cells were treated with lanthanum (La^3+^), a nonselective cation inhibitor. La^3+^ prevented the increase in intracellular Ca^2+^ (Figure 2(d), *n* = 86 cells). In addition, nifedipine, an inhibitor of voltage dependent L-type Ca^2+^ channels [24], has no effect (Figure 2(e), *n* = 59 cells). These results indicate that PM_10_ spontaneously triggers Ca^2+^ influx into the cytosol from the extracellular media, independent of voltage dependent L-type Ca^2+^ channels.

### 3.3. PM_10_-Induced Ca^2+^ Signal Is Associated with PLC/IP_3_ Pathway

**Figure 3:**
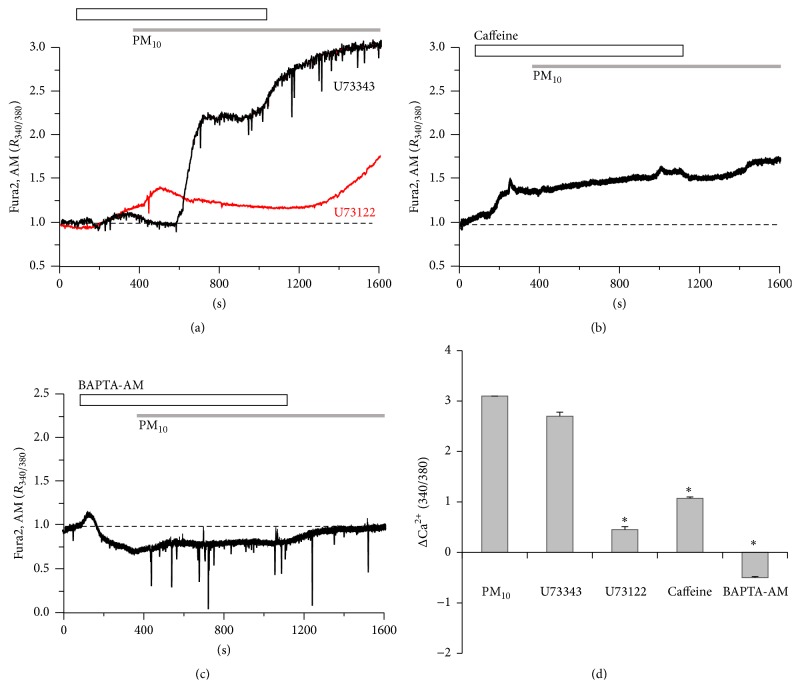
PM_10_-induced Ca^2+^ signaling is associated with the PLC/IP_3_ pathway. (a) 50 *μ*g/mL PM_10_-induced Ca^2+^ signal in the presence of PLC inhibitor 5 *μ*M U73122 or its inactive analog 5 *μ*M U73343. (b) The 50 *μ*g/mL PM_10_-induced Ca^2+^ signal was prevented by 20 mM caffeine, IP_3_R antagonist. (c) The 50 *μ*g/mL PM_10_-induced Ca^2+^ signal was completely abolished by 10 *μ*M BAPTA, AM. The top bars on trace panels indicate the type of extracellular solutions applied to the cells. (d) Data are represented as mean ± SE. ^*∗*^ *P* values <0.01 were considered significant. All traces are the reading from an experimental average. The dotted line shows the baseline at ratio 1.

To determine the role of the phospholipase C (PLC)/inositol 1,4,5-trisphosphate (IP_3_) pathway in PM_10_-induced Ca^2+^ influx, cells were pretreated with U73122, a specific blocker of PLC, or its inactive analog U73343, as a control. U73122 blocked PM_10_-induced Ca^2+^ increases (Figure 3(a), *n* = 135 cells), whereas U73343 did not prevent Ca^2+^ increases (Figure 3(a), *n* = 81 cells). After removal of U73122, the PM_10_-mediated signal increased. The PM_10_-induced Ca^2+^ signal was measured after treatment with 20 mM caffeine as an IP_3_ receptor (IP_3_R) antagonist [25] for 4 min. Caffeine completely eliminated the influx of Ca^2+^ induced by PM_10_ (Figure 3(b), *n* = 162 cells). When treated with BAPTA, AM, the PM_10_-induced Ca^2+^ signal was blocked by the chelation of basal and increased Ca^2+^ (Figure 3(c), *n* = 117 cells). These results suggest that PM_10_-induced Ca^2+^ increases were associated with the PLC-IP_3_-IP_3_R pathway.

### 3.4. PM_10_-Induced Ca^2+^ Signal Is Attenuated by Blocking the Oxidative Pathway

**Figure 4:**
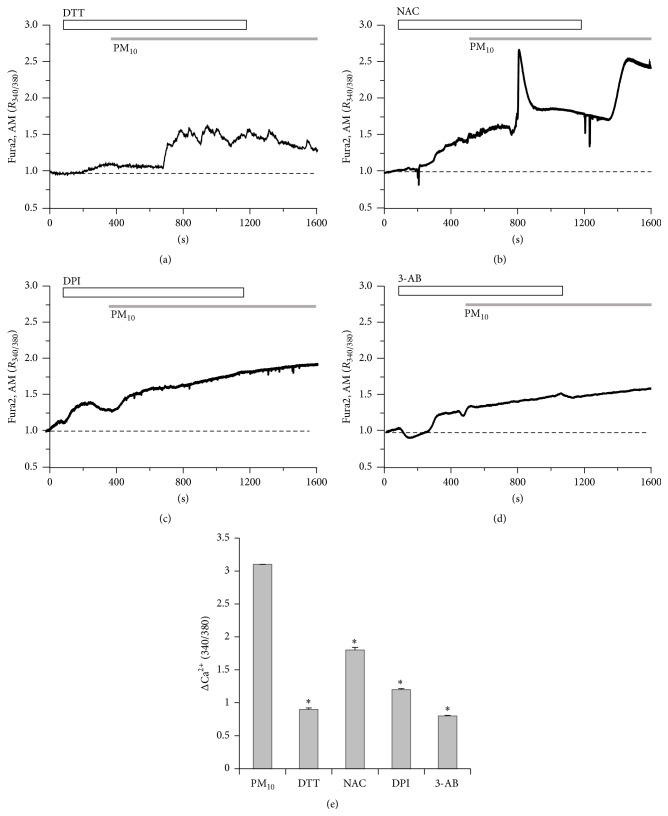
PM_10_-induced Ca^2+^ signal is attenuated by blocking the oxidative pathway. PM_10_ (50 *μ*g/mL) induced Ca^2+^ signaling in the presence of antioxidative compounds, (a) 1 mM DTT, (b) 5 mM NAC, and (c) 10 *μ*M DPI, and (d) in the presence of 10 *μ*M 3-AB, PARP-1 inhibitor. (e) Top bars on trace panels indicate the type of extracellular solutions applied to the cells. Data are represented as mean ± SE. ^*∗*^ *P* values <0.01 were considered significant. All traces are the reading from an experimental average. The dotted line shows the baseline at ratio 1.

Having established that ROS signal by PM_10_ in Figure 1 is needed for the alterations in airway epithelia, we then used several ROS scavengers on PM_10_-treated airway cells. To examine whether the PM_10_-induced Ca^2+^ increases were attributable to sulfhydryl oxidation-dependent mechanisms, cells were treated with DTT, a sulfhydryl-reducing agent [26]. Most of the PM_10_-induced oscillatory Ca^2+^ signals were diminished by DTT (Figure 4(a), *n* = 102 cells). PM_10_-induced Ca^2+^ signals were modestly attenuated by the antioxidant NAC (Figure 4(b), *n* = 174 cells), indicating that ROS signal is involved in the Ca^2+^ response. Moreover, PM_10_-induced Ca^2+^ increases were blocked by NADPH oxidase inhibitor DPI [27] (Figure 4(c), *n* = 141 cells). Although NAC and DPI attenuated the PM_10_-induced Ca^2+^ increases, the removal of NAC in the continued presence of PM_10_ again increased Ca^2+^. DPI-treated cells exhibited an increase in their basal Ca^2+^ level even though no transient increase in Ca^2+^ occurred, suggesting that NAC and DPI differ only in their ability to antagonize ROS-mediated Ca^2+^ release. To determine whether the PM_10_-induced response is involved in PARP signaling, we applied a well-characterized PARP-1 inhibitor, 3-AB [28]. 3-AB inhibited PM_10_-mediated Ca^2+^ increases markedly and protractedly (Figure 4(d), *n* = 182 cells). These results indicate that PM_10_-induced increases in Ca^2+^ in bronchial epithelial cells are mediated by oxidation and are dependent on PARP-1 signaling.

### 3.5. PM_10_-Induced Ca^2+^ Signal Is Required for the Involvement of TRPM2 Channel

**Figure 5:**
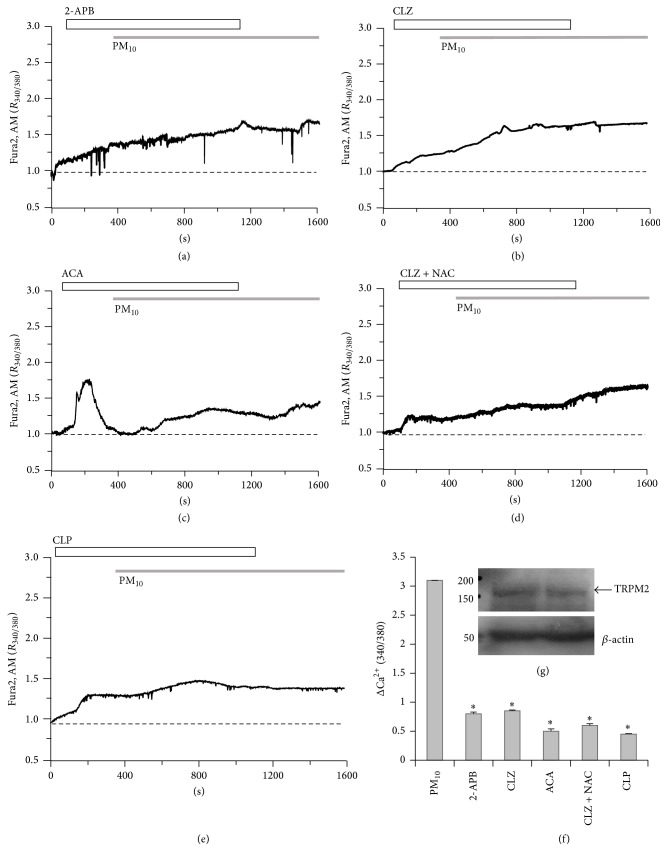
PM_10_-induced Ca^2+^ signaling requires TRPM2 channel. PM_10_ (50 *μ*g/mL) induced Ca^2+^ signaling in the presence of TRPM2 inhibitors: (a) 75 *μ*M 2-APB, (b) 10 *μ*M CLZ, (c) 10 *μ*M ACA, (d) 10 *μ*M CLZ + 1 mM NAC, or (e) 10 *μ*g/mL CLP. (g) TRPM2 protein was expressed in BEAS-2B cells and *β*-actin antibody was used as loading control. Duplicated samples were loaded. Top bars on trace panels indicate the type of extracellular solutions applied to the cells. Data are represented as mean ± SE. ^*∗*^ *P* values <0.01 were considered significant. All traces are the reading from an experimental average. The dotted line shows the baseline at ratio 1.

To determine whether the PM_10_-induced Ca^2+^ signal was mediated by TRPM2, cells were exposed to several types of TRPM2 inhibitors, 2-APB, CLZ, and ACA [29–34], before treatment with PM_10_. In our experiment, the application of 2-APB or CLZ (Figures 5(a) and 5(b), *n* = 75 and 110 cells, resp.), a well-characterized TRPM2 inhibitor, also significantly decreased PM_10_-induced Ca^2+^ influx in bronchial epithelial cells (Figures 5(a), 5(b), and 5(f)). ACA markedly decreased PM_10_-mediated influx; however, the initial Ca^2+^ peak was due to ACA by itself (Figures 5(c) and 5(f), *n* = 102 cells). To evaluate the combined effect of an antioxidant and TRPM2 blocker, cells were exposed to the antioxidant NAC and the TRPM2 inhibitor CLZ (CLZ + NAC^*∗*^) (Figures 5(d) and 5(f), *n* = 124 cells). PM_10_-induced Ca^2+^ increases were diminished by the combination treatment (Figures 5(d) and 5(f)). To determine if CaM mediated TRPM2 activation, cells were treated with CLP, a Ca^2+^-CaM inhibitor [35]. CLP markedly inhibited PM_10_-induced Ca^2+^ increase (Figures 5(e) and 5(f), *n* = 114 cells). We checked that BEAS-2B cells were expressed TRPM2 protein endogenously (Figure 5(g)). Taken together, these data suggest that the Ca^2+^ response observed in PM_10_-stimulated bronchial epithelial cells mediated by ROS/PARP/ADPR signaling to activate TRPM2.

### 3.6. The Modulation of PM_10_-Induced IL-8 mRNA Expression

**Figure 6:**
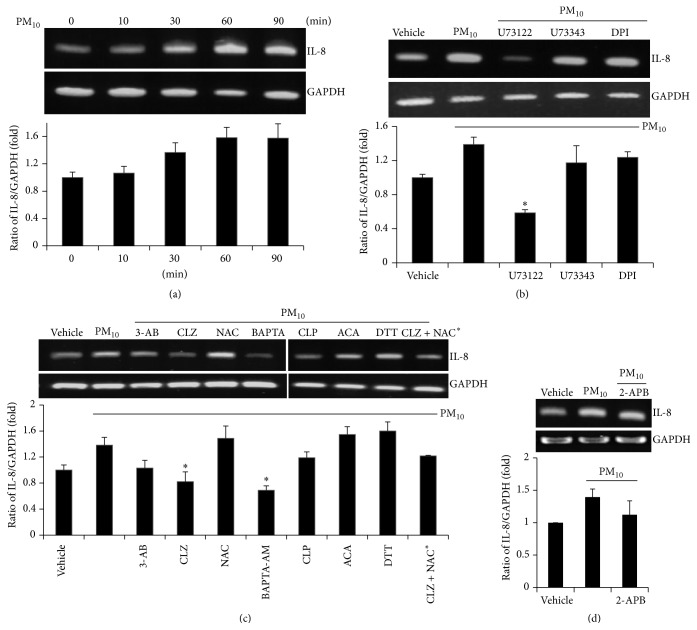
The modulation of PM_10_-induced IL-8 mRNA expression by several Ca^2+^ blockades. (a) PM_10_ treatment increased IL-8 mRNA expression. Cells were stimulated with 50 *μ*g/mL PM_10_ for the indicated time (*n* = 4). The total mRNA of stimulated cells was extracted and quantified after the value was normalized to GAPDH. (b–d) IL-8 mRNA expression treated with 50 *μ*g/mL PM_10_ for 90 min in the pretreatment of all compounds for 30 min, as used in Ca^2+^ measurement experiments (*n* = 4). The total mRNA of stimulated cells was extracted and quantified after the value was normalized to GAPDH and represented the ratio of IL-8/GAPDH (fold change to that of vehicle). Data are represented as mean ± SE. ^*∗*^ *P* values <0.01 were considered significant.

To determine whether PM_10_ induces the proinflammatory effect on bronchial epithelial cells, cells were treated with PM_10_ for different lengths of time to examine the expression level of IL-8. Expression of IL-8 mRNA levels was elevated after 30 min of treatment with PM_10_ (Figure 6(a), *n* = 4). Next, we assessed whether the blockades of Ca^2+^ signaling attenuate the expression of IL-8 mRNA level; cells were treated with PM_10_ after pretreatment with one of the following: the PLC inhibitor U73122, its inactive analog U73343, a NADPH oxidase inhibitor (DPI), a PARP-1 inhibitor (3-AB), a TRPM2 inhibitor (CLZ), an antioxidant (NAC), an intracellular Ca^2+^-selective chelator (BAPTA, AM), a Ca^2+^-CaM inhibitor (CLP), an anthranilic acid-derived TRPM2 inhibitor (ACA), a sulfhydryl-reducing agent (DTT), or a combined low dose of CLZ and NAC (CLZ + NAC^*∗*^). IL-8 mRNA levels were markedly decreased by U73122, 3-AB, CLZ, CLP, and the combination of CLZ and NAC (Figures 6(b) and 6(c), *n* = 4). U73343, which served as a positive control for U73122, did not prevent mRNA expression of IL-8. DTT and DPI did not affect PM_10_-induced expression of IL-8 mRNA, suggesting that sulfhydryl oxidation and NADPH oxidase-mediated effects may only partially affect PM_10_-induced signaling in bronchial epithelial cells, since PM_10_-induced Ca^2+^ signaling was attenuated by these compounds in Figures 4(a) and 4(c). As we predicted, a low dose of CLZ and NAC reduced IL-8 mRNA expression, indicating that this combination of compounds could be an efficient therapeutic strategy to treat dust particle-mediated airway disease. Treatment with 2-APB to block TRPM2 channel modestly attenuated PM_10_-induced IL-8 mRNA expression (Figure 6(d), *n* = 4). These data indicate that PM_10_ associated with the TRPM2-mediated Ca^2+^ increase, which, in turn, affected IL-8 mRNA expression in bronchial epithelial cells.

## 4. Discussion

In this study, we demonstrate the effect of several Ca^2+^ signaling blockades on dust particles PM_10_-mediated Ca^2+^ signaling and proinflammatory cytokine IL-8 mRNA expression in human bronchial epithelial cells. An increased ROS signal as well as plasma membrane channel activation caused by dust particles PM_10_ application can trigger intracellular Ca^2+^ and ROS signaling and the subsequent expression of proinflammatory cytokines. The enhanced ROS level by dust particles has been implicated in the pathology of several lung diseases, including asthma, lung fibrosis, and chronic obstructive pulmonary disease (COPD) [2, 36]. Moreover, changes in Ca^2+^ signaling are closely involved in various stress responses and the inflammatory response through activation of IL-8 production. It is therefore likely that the PM-induced Ca^2+^ signaling activates similar pathways to regulate the release of proinflammatory cytokines and may progress the immune response seen in asthma and COPD.

For the downstream of ROS signal, increased PARP activity facilitates ADPR synthesis [18]. The PARP activity is induced by the activation of TRPM2 upon oxidative stress, which may be caused by DNA damage [16, 29, 30]. The TRPM2 channel is activated by intracellular ADP-ribose (ADPR) and by several ROS messengers, leading to excessive influx of Ca^2+^ and other ions [18]. Moreover, intracellular or extracellular Ca^2+^ is known to be critical for TRPM2 activation [33] and mediated by Ca^2+^-saturated calmodulin (CaM) [34]. However, a TRPM2 deficiency model shows no anti-inflammatory effect in COPD which is associated with oxidative stress [37]. Recently, a negative feedback mechanism for TRPM2 was described in which ROS production is activated through inhibition of the membrane potential sensitive NADPH oxidase, thereby protecting the host against inflammation and tissue injury [38]. Our study reveals that dust particles promote consistent and excessive Ca^2+^ influx and provide the evidence that several blockades attenuate the signal through the activation of TRPM2, which regulates the expression of proinflammatory cytokines and may induce pathological responses beyond physiological homeostasis. Oxidative stress is associated with many pathologic events, including pulmonary fibrosis [39]. Indeed, the blockade of ROS signaling and TGF-*β*1/Smad2/3 exerts an antifibrotic activity in lung tissue [40]. Collectively, it appears that dust particles-mediated TRPM2 activation facilitates inflammatory events in COPD and pulmonary fibrosis patients. These findings are not surprising, considering the inflammatory role of dust particles in the airway epithelia. Airborne particles are well known to augment airway inflammation and exacerbate asthma symptoms by increasing IL-8 [41]. However, the attenuation of TRPM2 blockers in PM_10_-induced Ca^2+^ response and IL-8 expression will provide experimental relevance to apply in oxidative inflammatory lung diseases. Thus, the precise role of TRPM2 will be elucidated in pulmonary fibrotic tissues in the near future. Although neither NAC nor DPI are solely selective for ROS-dependent Ca^2+^ release and IL-8 mRNA expression induced by dust particles (Figure 4), these pharmacological agents can be used to reveal the specific involvement of ROS-mediated signaling by PM. Beyond increased ROS signal by PM, various chemical components of ambient dust particles mediating other signaling cascades might be triggered through other potential mechanisms including modulation of plasma membrane ion channel activity or endogenous enzyme activity [22].

Although dust particles PM_10_ was filtered with mesh which has pore size of 10 *μ*m, we observed that dust particles were also contained between 3 *μ*m and 100 nm in size (Figure 1(a)). This size of dust particles has the potential to assume the pathological role of fine nanoparticles, which can permeate deep into the lung and become incorporated into the airway epithelial cells and blood stream, mediating inflammatory reactions. Recently, size-dependent uptake and trafficking patterns of nanoparticles have been reported in the respiratory tract and immune system [42]. Despite their small size, previous studies have reported that dust particles contain biological and chemical materials capable of inciting serious airway inflammatory responses [36]. It is well known that most environmental particles contain endotoxin that contributes to various biological activities in epithelial and immune cells [36]. In this study, endotoxin contamination cannot be considered in current experimental condition because LPS concentration was below range (0.005 EU/mL) in heated PM. However, naturally originated dust particles with adhered microorganism or organic materials may mediate numerous functions and modulate additive signaling.

## 5. Conclusions

Dust particles induced intracellular Ca^2+^ signaling and proinflammatory cytokine IL-8 expression in human lung airway epithelial cells. Collectively, we suggest that the Ca^2+^ signaling by dust particles was attenuated by antioxidants, inhibitors of Ca^2+^ signaling pathway, and TRPM2 inhibitors and provides the evidence that treatment with blockades of Ca^2+^ signal should be considered for therapeutic trials in bronchial epithelia for inflammatory signaling caused by environmental dust events.

## Abbreviations

TRPM2: Transient receptor potential melastatin 2
ROS: Reactive oxygen species
PLC: Phospholipase C
IP_3_R: Inositol 1,4,5-trisphosphate receptor
PARP-1: Poly(ADP-ribose) polymerase 1
IL-8: Interleukin-8
BAPTA, AM: 1,2-Bis(2-aminophenoxy)ethane-N,N,N′,N′-tetraacetic acid tetrakis, acetoxymethyl ester
CLZ: Clotrimazole
3-AB: 3-Aminobenzamide
ACA: N-(p-Amylcinnamoyl)anthranilic acid
2-APB: 2-Aminoethoxydiphenyl borate
CLP: Chlorpromazine
DTT: Dithiothreitol
NAC: N-Acetylcysteine
DPI: Diphenyleneiodonium
CaM: Calmodulin.
